# Exploration of the mechanisms underlying the beneficial effect of Luo Tong formula on retinal function in diabetic rats via the “gut microbiota–inflammation–retina” axis

**DOI:** 10.1186/s13020-022-00688-3

**Published:** 2022-12-02

**Authors:** Sha Di, Chensi Yao, Liping Qiao, Xiuyang Li, Bing Pang, Jiaran Lin, Jia Wang, Min Li, Xiaolin Tong

**Affiliations:** 1 Department of Endocrinology, Guang’anmen Hospital, China Academy of Chinese Medical Sciences, Beijing, 100053 China; 2 Institute of Metabolic Diseases, Guang’anmen Hospital, China Academy of Chinese Medical Sciences, Beijing, 100053 China; 3 Clinical Medical College, Beijing University of Chinese Medicine, Beijing, 100029 China; 4 General Department, Guang’anmen Hospital, China Academy of Chinese Medical Sciences, Beijing, 100053 China; 5 Molecular Biology Laboratory, Guang’anmen Hospital, China Academy of Chinese Medical Sciences, Beijing, 100053 China

**Keywords:** Diabetic retinopathy, Luo Tong formula, Traditional Chinese medicine, Gut microbiota, Inflammation, Blood-retinal barrier

## Abstract

**Background:**

Diabetic retinopathy (DR) is a common microvascular complication of diabetes. Luo Tong formula (LTF), a classical traditional Chinese medicine (TCM) prescription, consists of four plants that have been widely and effectively used to treat DR. Previous work in our laboratory has confirmed that LTF can effectively ameliorate DR. However, the potential mechanism underlying the therapeutic effect of LTF on DR has not been fully elucidated. To explore the potential mechanism of action through which LTF prevents and alleviates DR from an inflammation and gut microbiota perspective.

**Materials and methods:**

Metabolite profiling of LTF was performed using liquid chromatography–mass spectrometry (LC–MS) and gas chromatography–mass spectrometry (GC–MS). Type 1 diabetes was induced in male Sprague Dawley (SD) rats via tail vein injection of 45 mg/kg streptozotocin. Next, 100 SD rats were randomly divided into four groups, normal control; diabetic control; diabetic + insulin + calcium dobesilate; and diabetic + insulin + LTF. After 12 weeks of treatment, glucose metabolism, fundus oculi, blood-retinal barrier permeability, retinal thickness, microvascular damage, as well as cell junction expression in retinas were measured and the changes observed in different groups were compared. Finally, the alteration in gut microbiota and inflammatory cytokine expression in serum and tissues were monitored, and their correlation was analyzed.

**Results:**

A total of 1024 valid peaks were obtained for LTF using GC–MS. The HbA1c and fasting blood glucose (FBG) levels in the LTF group were slightly decreased. LTF exerted protective effects on fundus oculi and the retina structure to different degrees. LTF attenuated systemic and local retinal inflammation by significantly decreasing the levels of seven pro-inflammatory cytokines, including ICAM-1, IL-6, IL-8, MCP-1, VCAM-1, VEGF, and IL-1β. LTF restored the intestinal microbiota of diabetic rats to levels that were similar to those of normal rats. Further analysis revealed that *Enterobacteriales*, *Prevotellaceae*, *Enterobacteriaceae*, *Bacteroides*, and *Klebsiella* were significantly and positively correlated with the inflammatory factors in DR after LTF treatment.

**Conclusions:**

Our results revealed the mechanisms underlying the preventive effects of LTF on DR development and progression. LTF inhibited pathological changes in retinal histopathology, cell composition, and cell junction proteins while effectively ameliorating systemic and local retinal inflammation via regulating pivotal gut microbiota.

## Introduction

The Diabetes Atlas (10th Edition) released by the International Diabetes Federation (IDF), reported that 537 million, 20–79 year old adults worldwide were living with diabetes in 2021 (https://diabetesatlas.org). Diabetic retinopathy (DR), a common microvascular complication of diabetes mellitus (DM), can trigger preventable blindness in the adult working population. It is predicted that the estimated 103.12 and 28.54 million adults suffering from DR and vision-threatening DR in 2020 will increase to 160.50 and 44.82 million in 2045, respectively [1].

Intra-retinal microvascular anomalies are an early clinical symptom of DR. Retinal blood vessels, complex multicellular units, consist of endothelial cells, pericytes, glia, neuronal processes, and other cells. These neurovascular units regulate the integrity of the blood-retinal barrier (BRB), a highly selective entity that protects the retina from possibly harmful molecules by regulating the molecular exchange between the retina and the circulatory system. The BRB includes inner and outer components consisting of tight junctions between adjacent pigmented epithelial cells. Retinal vascular endothelial cells are an important part of the inner BRB. Pericytes, which regulate endothelial cell function and the expression of tight junction proteins, provide structural support to wall vessels. Furthermore, disruption of BRB and cell junction results in elevated retinal permeability, thus allowing the occurrence of early stages DR [2].

Chronic inflammation is a crucial factor in DR progression. Elevated levels of inflammatory cytokines [tumor necrosis factor-alpha (TNF-α), interleukin-1β (IL-1β), interleukin-6 (IL-6), interleukin-8 (IL-8), and nuclear factor-κB (NF-κB)], cell adhesion molecules [intercellular adhesion molecule 1 (ICAM-1) and vascular cell adhesion molecule-1 (VCAM-1)], chemotactic proteins [monocyte chemoattractant protein-1 (MCP-1)], growth factors [vascular endothelium growth factors (VEGF)], and bioactive molecules affect endothelial cells as well as pericytes, damage BRB, and increase vascular permeability; thus, ultimately accelerating the occurrence and progression of DR [3]. In diabetic patients, increased TNF-α and IL-1β levels represent potent positive upregulators of ICAM-1 in endothelial cells. NF-κB activation in diabetes is followed by an increase in the levels of cytokines, adhesion molecules, VCAM-1, and VEGF. VEGF facilitates pathological angiogenesis and enhances vascular permeability; thus representing a major therapeutic target in DR [4]. Chronic inflammation, which occurs during the development of DR, was recently discovered to be associated with gut microbiota. Hyperglycemia leads to gut microbiota dysbiosis and gut barrier alterations, resulting in a systemic, low-grade chronic inflammatory situation which disrupts immune homeostasis. These factors lead to retinal inflammation, BRB breakdown, and neovascularization [5]. Altered phyla, including those of *Bacteroidetes* (45.8%) and *Firmicutes* (23.8%), among others, were associated with DR characteristics such as acellular capillaries, seen in diabetic mice. T1DM patients have been shown to display higher concentrations of serum inflammatory cytokines, including TNF-α, IL-6, and IL-1; thus suggesting that chronic inflammation initiates at the onset of diabetes and exists until the DR occurrence [5].

Calcium dobesilate (CaD), a vasoactive and angio-protective drug which significantly alleviates DR symptoms, is widely used to treat this disease [6]. CaD reduced vascular leakage by inhibiting the VEGF-activated p38 MAPK pathway in diabetes patients [7]. In diabetic rats, CaD reduced vascular injury and DR progression by attenuating vascular tortuosity, acellular capillaries, and pericyte loss [8]. Furthermore, the antioxidant properties of CaD helped reverse retinal pro-inflammatory processes in diabetic rats [9].

**Fig. 1:**
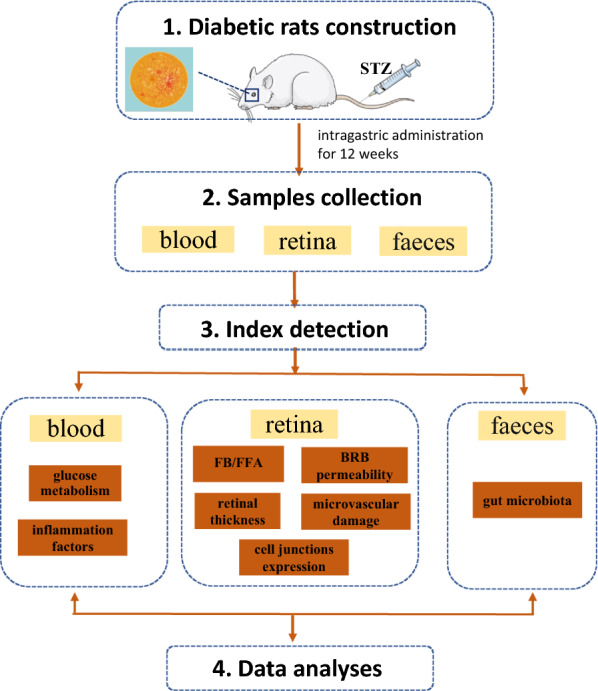
The whole framework of this study

Luo Tong Formula (LTF), changed from the Di Dang Tang formula, which was documented in Shanghan Zabing Lun written by Zhang Zhongjing in 200–210 A.D during the East Han Dynasty. Di Dang Tang has effect on eliminating stagnated heat and treating stagnated blood syndrome [10]. LTF is composed of Rhei Radix Et Rhizoma (Chinese name “Da Huang”), Persicae Semen (Chinese name “Tao Ren”), Notoginseng Radix Et Rhizoma (Chinese name “San Qi”), and Hirudo (Chinese name “Shui Zhi”). Rhei Radix Et Rhizoma is dried roots and rhizomes of *Rheum palmatum* L., *Rheum tanguticum Maxim. ex* Balf., or *Rheum officinale* Baill. Persicae Semen is dried seeds of *Prunus persica* (L.) Batsch or *Prunus davidiana* (Carr.) Franch. Notoginseng Radix Et Rhizoma is dried roots and rhizomes of *Panax notoginseng* (Burk.) F. H. Chen. Lastly, Hirud is dried *Whitmania pigra* Whitman, *Hirudo nipponica* Whitman, or *Whitmania acranulata* Whitman. LTF is mainly used to treat vascular lesions with blood stasis and toxin block collaterals [11], as Rhei Radix Et Rhizoma, Persicae Semen, Notoginseng Radix Et Rhizoma, and Hirudo are effective in removing blood stasis and promoting blood circulation [12]. Previous studies have shown that LTF protects retinal micro-vessel kinetic morphological changes and improves abnormal hemorheology in diabetic rats [11, 12]. Furthermore, LTF prevents DR via targeting the Micro-200b and the p38MAPK/NF-κB pathway [13, 14]. However, the association between this mechanism and gut microbiota remains unclear. In this study, LTF was used to treat T1DM rats via gavage for 12 weeks, following which, changes in glucose levels, retinal histopathology, inflammation, BRB, and gut microbiota were assessed to determine whether LTF prevents the development of DR via the “microbiota–inflammation–retina” axis (Fig. 1).

## Materials and methods

### Reagents

LTF, consisting of *Radix Rhei Et Rhizome* (18070049), *Persicae Semen* (19040204), *Panax Notoginseng (Burk.) F. H. Chen Ex C. Chow* (1906075), and *Hirudo* (18050160) was obtained from the Sichuan New Green Pharmaceutical Technology Company (Chengdu, China). Recombinant spermine zinc insulin injections (Batch No. 21905202) and CaD capsules (Registration Certificate No. of imported drugs: H20140641) were purchased from Jiangsu Wanbang Biochemical Medicine Company (Jiangsu, China) and EBEWEPharma GmBH Nfg. KG (Unterach, Austria), respectively.

### Animals

Male, 8-week old SD rats (Beijing Weitong Lihua Experimental Animal Technology Company, Beijing, China) were bred at the Institute of Radiology, Chinese Academy of Medical Sciences, Nankai District, Tianjin. All animals had free access to food and water and were housed at room temperature of 22 ± 2 °C with a relative humidity of 40–60%. The protocol was approved by the Animal Protection and Use Committee of the Institute of Radiology, Chinese Academy of Medical Sciences with approval No. IRM-DWLL-20200084.

### Experimental design

Following an acclimatization period of 1 week, 25 of the 100 SD rats were randomly divided into the blank control group. Next, 100 SD rats fasted for 12 h, then STZ (45 mg/kg, 0.1 mol/L citric buffer, pH 0.5) was injected intravenously. The normal control group was injected with citric buffer. Random blood glucose was measured in blood samples collected from the tail vein on three consecutive days. Rats with blood glucose < 16.7 mmol/L were injected with STZ again and blood glucose was measured again on day 7. The rat diabetic model was considered to have been successfully established if the blood glucose was ≥ 16.7 mmol/L. All diabetic rats were randomly divided into three groups, (1) M group (n = 25), fed with a similar amount of intra-gastric administration of distilled water; (2) CaD group (n = 25), given a gavage of CaD (0.25 g/kg) and injected with insulin (1.5 IU/time/day) intramuscularly; (3) LTF group (n = 25), given a gavage of LTF (2 g/kg) and intramuscularly injected with insulin (1.5 IU/time/day). Normal control rats (N group), were subjected to intragastric administration of the same amount of distilled water. All rats were treated with drugs or distilled water once daily for 12 weeks.

The treatment lasted for 12 weeks, after which all animals were weighed and anesthetized with chloral hydrate. Stool samples were collected prior to anesthesia. The following specimens were collected: (1) blood; (2) serum (abdominal aorta blood was collected and centrifuged to separate the serum); and (3) rat retina.

### LTF metabolite profiling using LC-MS

Accurately weighed LTF 10 daily prescriptions of granules, which composed of Rhei Radix Et Rhizoma 10 g, Persicae Semen 50 g, Hirudo 30 g, Notoginseng Radix Et Rhizoma powder 15 g and dissolved in 600 mL water. The sample was centrifuged and supernatant (200 μL) was taken. 20 μL of l-2-chlorophenyl alanine (0.3 mg/mL) dissolved in methanol as internal standard, and an 800 μL mixture of methanol and water (1/4, vol/vol) was added to each sample, and the tube was vortexed for 2 min. Ultrasonic extraction of all samples in the ice water bath for 30 min, and placed at − 20 °C for 2 h. Subsequently, the samples were centrifuged at 4 °C (13,000 rpm) for 10 min. The supernatants (150 μL) from each tube were filtered via 0.22 μm microfilters and transferred to LC vials for LC–MS analysis. Both electrospray ionization (ESI)-positive and ESI-negative ion modes of metabolites were analyzed using a Dionex Ultimate 3000 RS UHPLC (Thermo Fisher Scientific, Waltham, MA, USA). All ion modes were separated by an ACQUITY UPLC HSS T3 column (1.8 μm, 2.1 × 100 mm). A detailed description of the protocol was previously published [15].

### LTF metabolite profiling using GC-MS

Until the samples were centrifuged at 4 °C (13,000 rpm) for 10 min, the sample preparation method for GC–MS was the same as that for LC–MS. Nextly, the supernatants (150 μL) were put into glass vials and dried by concentration centrifugal dryer. 80 μL of 15 mg/mL methoxylamine hydrochloride pyridine solution was added to the glass vials, then the mixture was vortexed for 2 min and incubated at 37 °C for 90 min. 80 μL of BSTFA (with 1% TMCS) and 20 μL n-hexane were added into the mixture. 10 μL of C8, C9, C10, C12, C14, C16 (0.16 mg/mL) and C18, C20, C22, C24, C26 (0.08 mg/mL) were added as internal standards. The mixture was vortexed for 2 min and derivatized at 70 °C for 60 min. After the samples were removed, they were placed at room temperature for 30 min for GC–MS analysis. The LTF sample metabolites were measured and analyzed using an Agilent 7890 B GC + 5977A MSD (Agilent Technologies Inc., Santa Clara, CA, USA). DB-5 ms GC columns (30 m × 0.25 mm × 0.25 μm, Agilent J & W Scientific, Folsom, CA, USA) were used to isolate compounds. The flow rate of helium (> 99.999%), as the carrier gas, was 1 mL/min. The detailed protocol has been previously presented [15].

### Fundus photography (FP) and fundus fluorescein angiography (FFA)

Before anesthesia with 10% chloral hydrate (0.3 mL/0.1 kg), select rats were weighed. The right eye of each rat was dilated with compound tropicamide and then dripped with obucaine hydrochloride for anesthesia. Color FP was performed when the color of the right eye adjusted. Next, fluorescein sodium (60 µL) was injected into the tail vein for FFA examination. The fundus was observed using the Micro III retinal imaging system (Phoenix Research Labs, USA).

### Measurement of BRB permeability

Rats BRB permeability was measured using Evans blue (EB), which was injected into the tail vein (3% EB; 45 mg/kg) and allowed to diffuse for 2 h. Next, the pleural cavity was opened and a needle was inserted into the aortic arch from the left ventricle. Citric acid buffer at 37 °C was continuously injected into rats until the livers turned white. The eyeballs were collected and the retinas were immediately extracted. The retinas were dried at 90 °C for 45 min in an oven to obtain the dry weight. Next, retinas were placed in 120 μL formamide at 70 °C for 18 h to extract the EB. Subsequently, the supernatants were obtained from retinas centrifuged for 45 min at 15,000 rpm. The absorbance of the supernatants was measured at 620 and 740 nm using a microplate reader (Meilin Hengtong, China). Finally, the concentration of EB was assessed according to the standard curve of EB in formamide. EB leakage = actual concentration × 120 µL. The final result according to this formula was calculated as ng/mg = EB leakage/dry weight of retinas.

### Retinal thickness

Fresh eyeballs were preserved in 4% paraformaldehyde for 24 h and then embedded in paraffin. A detailed protocol has been previously described [15]. Finally, the slices were imaged using a light microscope (BX41 microscope; Olympus, Otsu, Japan). Retinal thickness was estimated using the obtained images and ImageJ (NIH, Bethesda, MD, USA).

### Trypsin digestion test of retinas

Eyeballs were stored with 4% paraformaldehyde for 24 h, following which the retinas were extracted. Next, the retinas were placed in a pepsin solution at 37 °C for 1 h and trypsin solution at 37 °C for 3 h. Following dehydration, retinas were stained using periodic acid-Schiff (PAS) and imaged using a light microscope.

### Western blot

The protein concentrations from retinas were quantified using a BCA assay kit (Cwbiotech, China). Proteins were separated via sodium dodecyl sulfate–polyacrylamide gel electrophoresis and transferred onto nitrocellulose membranes (Millipore, Billerica, MA, USA). The membranes were sealed with blocking buffer containing 5% non-fat milk for 1 h at room temperature and incubated with primary antibodies at 4 °C overnight on a shaker. The primary antibodies included rabbit anti-ZO-1 (1:1000, Proteintech, Chicago, IL, USA), rabbit anti-occludin (1:1000, Abcam, Cambridge, UK), rabbit anti-Claudin-5 (1:1000, Abcam), rabbit anti-VE-cadherin (1:1000, Abcam), rabbit anti-NF-KB (1:1000, Abcam), and rabbit anti-TNF-α (1:1000, Abcam). Then, the membranes were incubated with goat anti-rabbit IgG (H+L) -HRP (1:10,000) secondary antibodies (Jackson, USA) for 40 min at room temperature. Enhanced chemiluminescence (Millipore) was used to visualize bound antibodies and images were analyzed using the Gel Image system ver.4.00 (Tanon, China). GAPDH served as an internal reference for standardization.

### RT-PCR

The RNA was extracted from the retina using a TRIzol kit (Invitrogen, Carlsbad, CA, USA). The total RNA was reverse transcribed using the HiFiScript first-strand cDNA synthesis kit (Cwbiotech) at 37 °C for 40 min and at 70 °C for 10 min. RT-PCR was performed using a SYBR PCR mixture and the process included pre-incubation at 95 °C for 10 min, amplification (45 cycles) at 95 °C for 10 s, 59 °C for 60 s, melting curves at 95 °C for 15 s, 72 °C for 15 s, and 95 °C for 15 s. Eventually, reaction samples were prepared by mixing 10 µL SYBR Master Mix (2×) Universal, 1.5 µL Primer F (10 μM), 1.5 μL Primer R (10 μM), 3 µL template, 0.5 µL ROX correction dye, and H_2_O (final volume, 20 µL). Relative gene expression was evaluated using the 2^−ΔΔCT^ method.

### 16S rRNA gene sequencing

Fecal DNA was obtained using a QIAamp DNA Stool Mini Kit (Qiagen, Hilden, Germany). The V3–V4 regions of the 16S rRNA gene were amplified via PCR utilizing standard primers. The primers included Illumina 5′ overhang adapter sequences for two-step amplicon library building, following the manufacturer's instructions for overhang sequences and barcodes. Barcoded PCR products were purified and quantified using a DNA gel extraction kit (Axygen, Union City, CA, USA) and an FTC-3000 TM real-time PCR system (Funglyn Shanghai, China), respectively. PCR products were blended at equal ratios. Next, dual 8-bp barcodes were used for multiplexing. After eight PCR cycles, two unique barcodes were merged at either end of amplicons. The library was purified and sequenced using the Illumina Novaseq 6000 platform and 250-bp paired-end reads by TinyGen Bio-Tech (Shanghai, China) Co., Ltd.

Raw Fastq files were demultiplexed according to the used barcodes. Low-quality base pairs were eliminated from PE reads, according to the following condition (SLIDINGWINDOW: 50:20 MINLEN: 50). Fastq sequences were combined adopting FLASH program software (version 1.2.11) with default parameters. Low-quality contigs were eliminated according to the screen.seqs command with the condition. Data were analyzed with several software, including mothur (version 1.33.3), UPARSE (usearch version v8.1.1756, http://drive5.com/uparse/), and R (version 3.6.3). Operational taxonomic units (OTUs) were obtained after reads were clustered in a 97% sequence. OTU representative sequences contrasted with certain databases, including Silva 128, following the use of mothur (classify.seqs) software. Finally, the remaining OTUs were used for later analysis, including phylum, class, order, family, genus, and species.

### Measurement of glucose metabolism and serum cytokines

Glucose metabolism indicators, including HbA1c and FBG levels, were detected using a biochemical analyzer. Serum inflammatory parameters, including ICAM-1, IL-6, IL-8, MCP-1, VCAM-1, VEGF, and IL-1β were measured using ELISA kits.

### Statistics

Data analyses were performed using the IBM SPSS Statistics 26 (Chicago, IL, USA). Data are expressed as mean ± standard deviation. Statistical analyses, including t-tests, one-way ANOVA, and nonparametric tests were performed as appropriate, depending on the conditions. For gut microbiota data, the Kruskal–Wallis test was used to compare between the groups. Kruskal–Wallis test was performed using the “*ggpubr*::compare_means” function from the “*ggpubr*” package in R to measure the significant changes in α diversity between different groups. Based on the Bray Curtis distance, analysis of similarity (ANOSIM), and principal coordinate analysis (PCoA), which compare between group similarities, were performed using the “*vegan*” and “ape” package in R. Spearman’s correlation coefficient was used to analyze the relationships between groups.

## Results

### LTF metabolite profiling using LC-MS and GC-MS

**Fig. 2:**
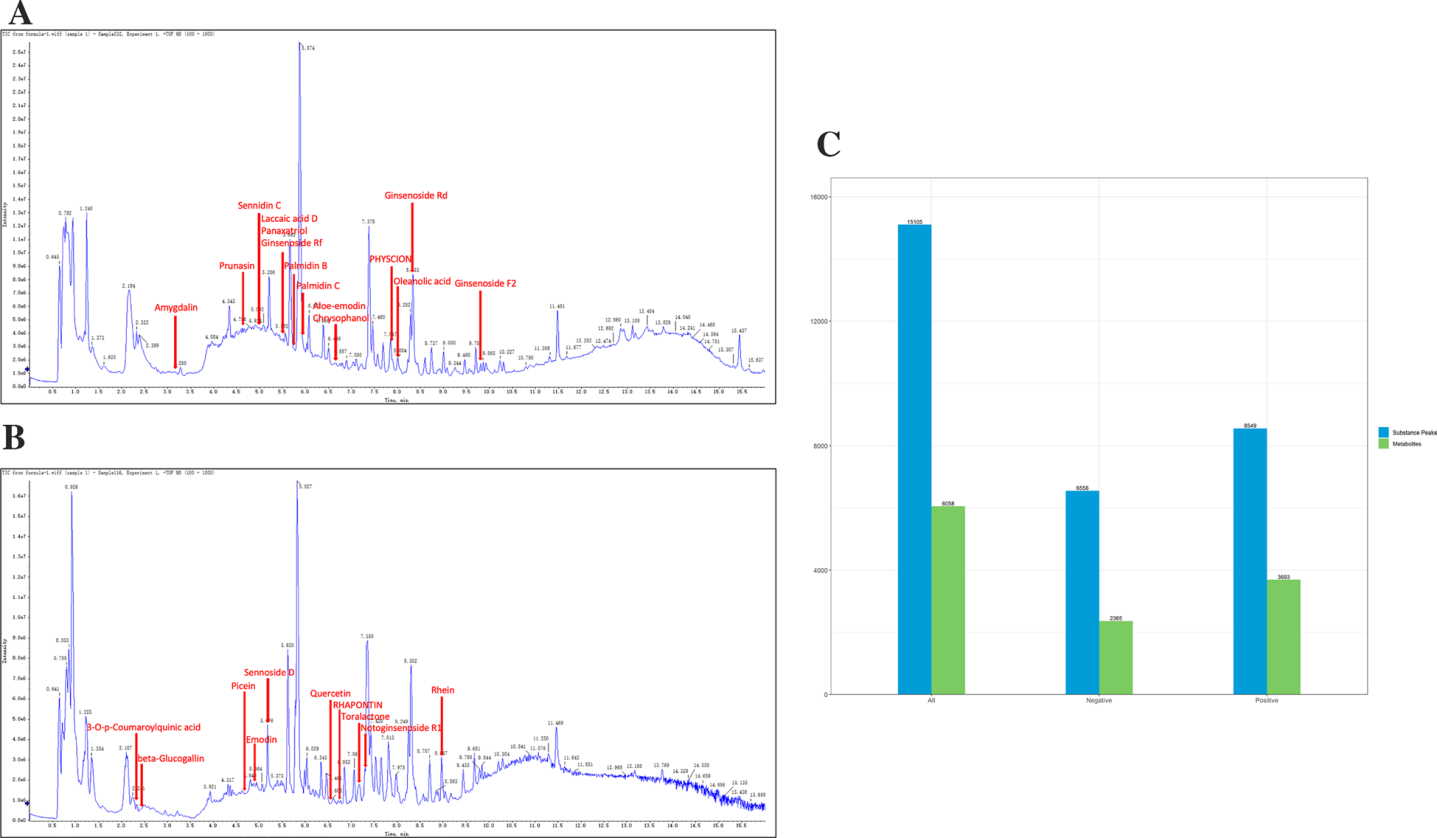
Total ions chromatogram (TIC) of water extracts from LTF. **A** TIC of positive ion model, **B** TIC of negative ion model, **C** the number of substance peaks determined, and metabolites identified

LC–MS and GC–MS were used to analyze and evaluate the LTF quality. The composition of LTF is listed in Table 1. A total of 15,105 substance peaks were identified in the LTF using LC–MS. A total of 6058 metabolites corresponding to the 15,105 substance peaks were detected, including 2365 metabolites revealed by the negative ion mode (Fig. 2A) and 3693 metabolites revealed by the positive ion mode (Fig. 2B, C). The main metabolites are summarized in Table 2. A total of 1024 valid peaks were detected in LTF, of which 291 were obtained after filtering, denoising, removing redundancies, and combining peaks. Firstly, we queried the main ingredients of each herb in the Chinese Pharmacopoeia and TCMSP database and merged them. Subsequently, the results of metabolites detected by MS were compared to find the overlapping components. Finally, metabolites in LTF were determined using the following metabolomics databases: the Human Metabolome Database (HMDB), Lipidmaps (v2.3), and METLIN.

**Table 1:** LFT composition

Species	Herbal name	Used part	Dosage (g)
*Rheum officinale* Baill*.*; *Rheum palmatum* L.; *Rheum tanguticum* Maxim.ex Balf	*Rhei radix et rhizoma*	Root and rhizoma	1
*Prunus persica* (L.) Batsch; *Prunus davidiana* (Carr.) Franch	*Persicae semen*	Seed	5
*Panax notoginseng* (Burk.) F. H. Chen	*Notoginseng radix et rhizoma*	Root	3
*Whitmania pigra* Whitman; *Hirudo nipponica* Whitman; *Whitmania acranulata* Whitman	*Hirudo*	Dry body	3

**Table 2:** Characterization of LTF metabolites

Peak no.	Metabolites	Formula	Source
1	beta-Glucogallin	C_13_H_16_O_10_	*Rhei radix et rhizoma*
2	Chrysophanol	C_15_H_10_O_4_	*Rhei radix et rhizoma*
3	Ginsenoside Rd	C_48_H_82_O_18_	*Notoginseng radix et rhizoma*
4	Ginsenoside Rf	C_42_H_72_O_14_	*Notoginseng radix et rhizoma*
5	Amygdalin	C_20_H_27_NO_11_	*Persicae semen*
6	Prunasin	C_14_H_17_NO_6_	*Persicae semen*
7	Sennoside D	C_42_H_40_O_19_	*Rhei radix et rhizoma*
8	Aloe-emodin	C_15_H_10_O_5_	*Rhei radix et rhizoma*
9	Oleanolic acid	C_30_H_48_O_3_	*Notoginseng radix et rhizoma*
10	Toralactone	C_15_H_12_O_5_	*Rhei radix et rhizoma*
11	Ginsenoside F2	C_42_H_72_O_13_	*Notoginseng radix et rhizoma*
12	Panaxatriol	C_30_H_52_O_4_	*Notoginseng radix et rhizoma*
13	Laccaic acid D	C_16_H_10_O_7_	*Rhei radix et rhizoma*
14	Rhein	C_15_H_8_O_6_	*Rhei radix et rhizoma*
15	Palmidin C	C_30_H_22_O_7_	*Rhei radix et rhizoma*
16	PHYSCION	C_16_H_12_O_5_	*Rhei radix et rhizoma*
17	Notoginsenoside R1	C_47_H_80_O_18_	*Notoginseng radix et rhizoma*
18	Sennidin C	C_30_H_20_O_9_	*Rhei radix et rhizoma*
19	Emodin	C_15_H_10_O_5_	*Rhei radix et rhizoma*
20	Palmidin B	C_30_H_22_O_7_	*Rhei radix et rhizoma*
21		C_15_H_10_O_7_	*Notoginseng radix et rhizoma*
22	3-*O*-*p*-Coumaroylquinic acid	C_16_H_18_O_8_	*Persicae semen*
23	RHAPONTIN	C_21_H_24_O_9_	*Rhei radix et rhizoma*
24	Picein	C_14_H_18_O_7_	*Notoginseng radix et rhizoma*

### LTF treatment affected glucose metabolism in a rat model

**Fig. 3:**
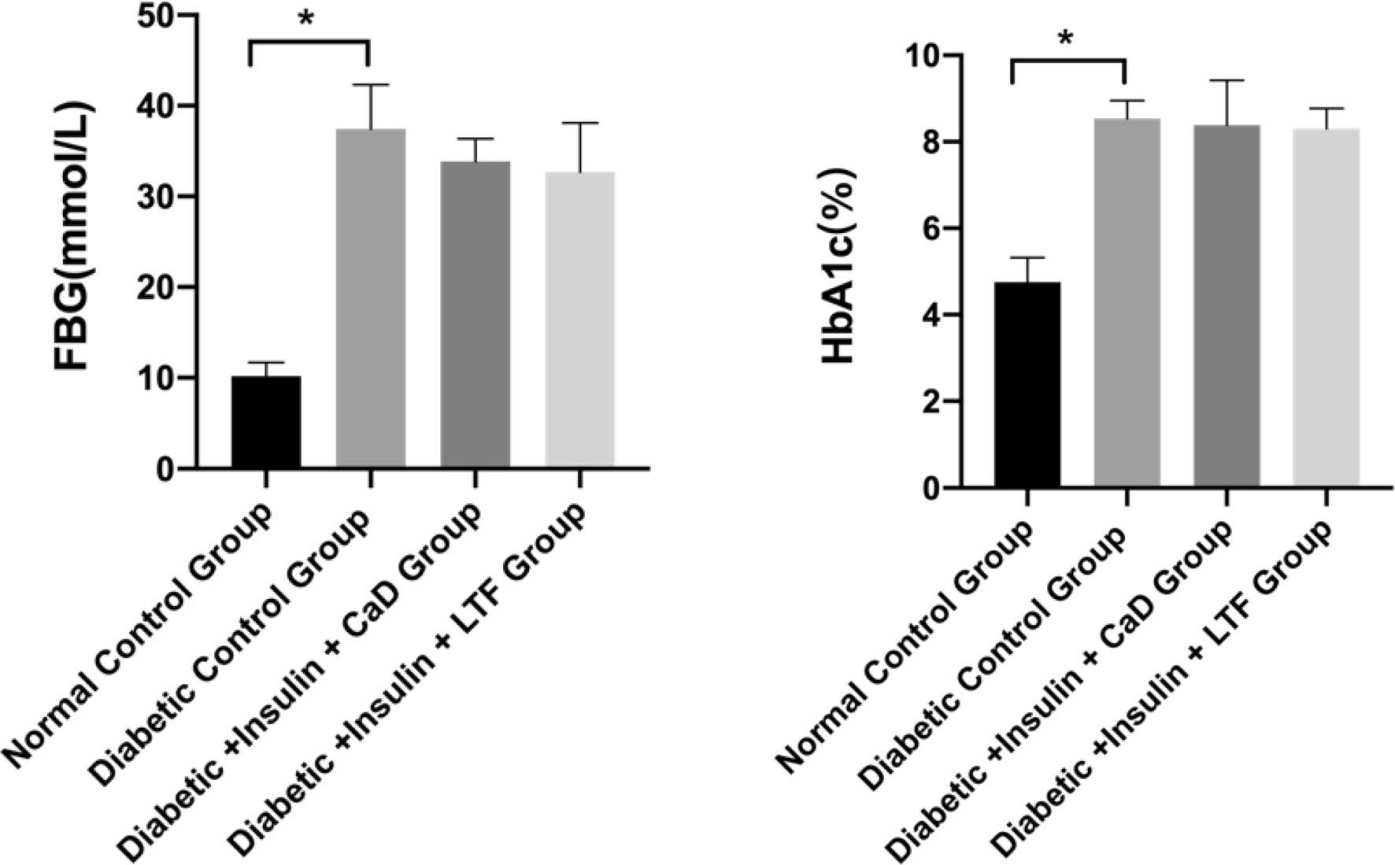
Effect of LTF and CaD on glucose metabolism. Data are expressed as mean ± SD (n = 6) (*p < 0.05: vs. normal control group; ^△^*p* < 0.05: vs. diabetic control group)

To investigate the impact of different treatments on diabetic rats, we compared the differences in blood glucose levels after 12 weeks of treatment. The levels of HbA1c and FBG in the diabetic control group (M group) were significantly higher than those in the normal control group (N group) (*p* < 0.001). The HbA1c and FBG levels of diabetic + insulin + CaD group (CaD group) and diabetic + insulin + LTF group (LTF group) were slightly decreased (*p* > 0.05) compared to those observed in rats from the M group (Fig. 3).

### LTF treatment affected fundus oculi changes in a rat model

**Fig. 4:**
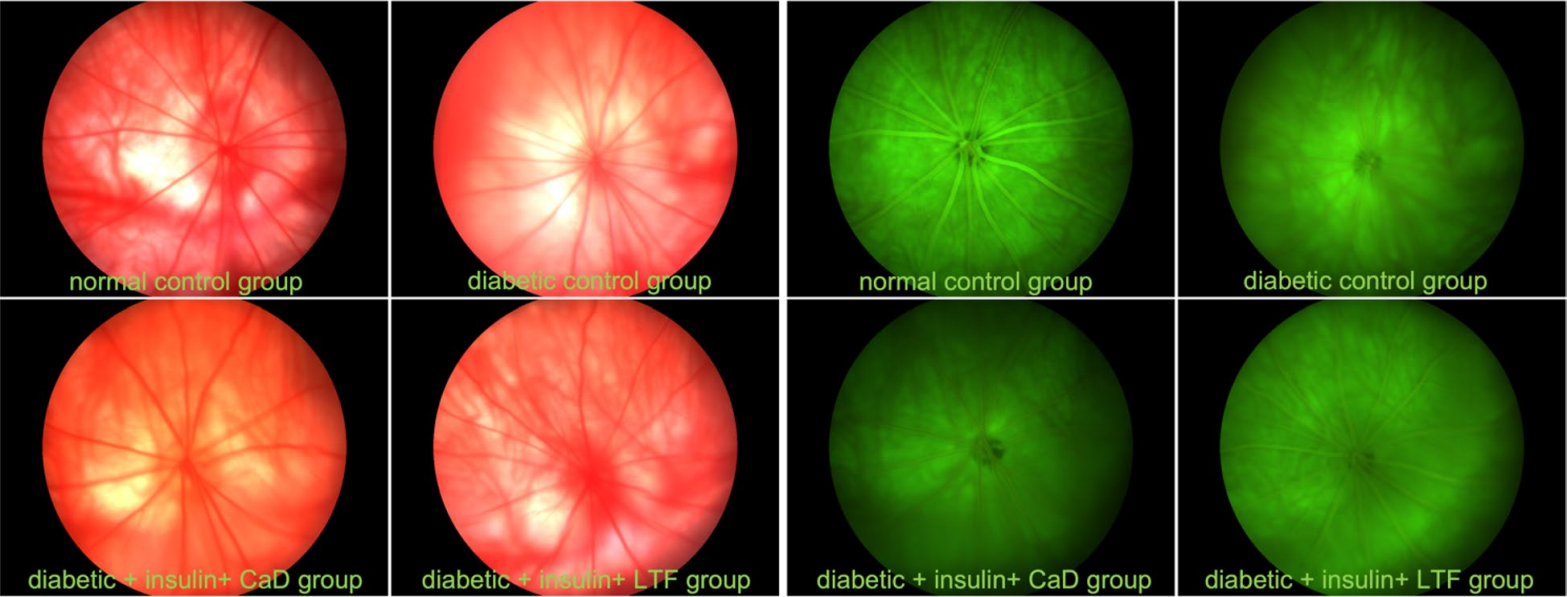
Effect of LTF and CaD on the fundus oculi changes observed by FP and FFA examinations

Retinal vasculature was observed using FP and FFA. In normal rats, FP and FFA results indicated a normal arteriovenous ratio, as well as clear fundi. Compared to normal rats, the FPs of diabetic ocular fundi showed yellowish-white spots, namely hyperfluorescence. Upon FFA examination, contiguous patches, retinal veins with widened diameters, and smaller arteriovenous ratios were observed. The yellowish-white spots, adjacent patches, arteriovenous ratios, and hyperfluorescence observed in diabetic rats, were significantly attenuated following CaD and LTF treatments (Fig. 4). Compared with the CaD treatment, the LTF treatment exerted a greater effect on fundus oculi changes.

### LTF treatment decreased BRB permeability and increased retinal thickness in a rat model

**Fig. 5:**
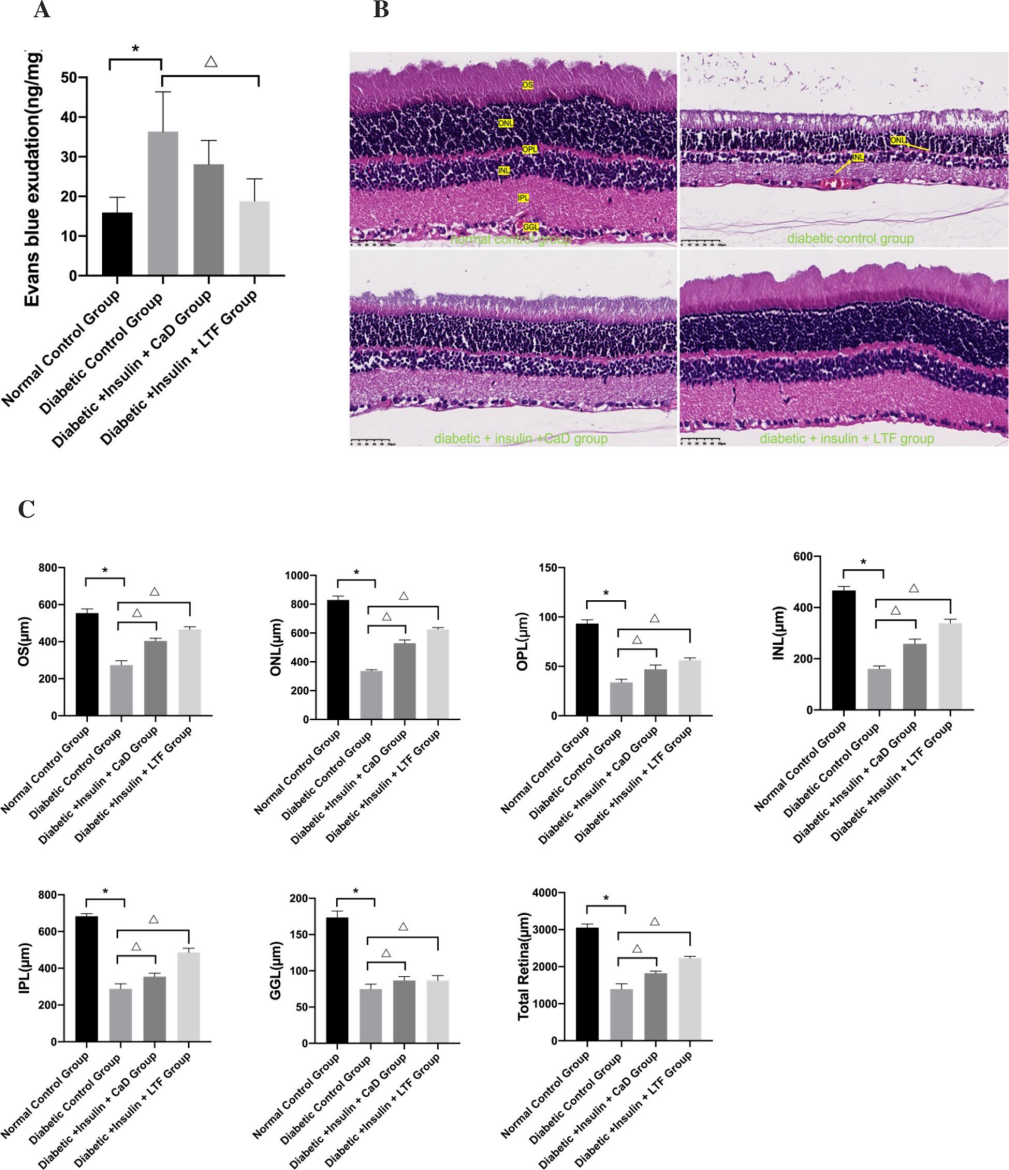
Effect of LTF and CaD on the BRB permeability and retinal thickness. **A** Evans blue exudation of different groups. **B** HE staining of retina (×400). **C** The retinal thickness. Data are expressed as mean ± SD (n = 3–4) (*p < 0.05: vs. normal control group; ^△^*p* < 0.05: vs. diabetic control group)

Hyperglycemia significantly increased BRB permeability in diabetic rats compared to that in normal rats. Furthermore, the BRB permeability of the LTF treated diabetic rats was significantly decreased compared to that of control diabetic rats. Although BRB permeability in the CaD group seemed to have decreased, this difference was not significant (Fig. 5A).

H&E staining was used to observe the retinal histopathological change. Rats in the normal control group had clear retinal structure and an orderly arrangement of cells in each layer, whereas the cells were arrayed disordered in diabetic rats. CaD and LTF treatment alleviated the retinal histopathological damage, including improved retinal structure and cell organization (Fig. 5B). The thickness of the photoreceptor outer segment (OS), outer nuclear layer (ONL), outer plexiform layer (OPL), inner nuclear layer (INL), inner plexiform layer (IPL), and ganglion cell layer (GGL), and the total retina, were determined via H&E staining. Compared with the normal control group, the thickness of each retinal layer in the diabetic control group was significantly reduced. CaD and LTF treatment dramatically increased retinal thickness compared to that measured in the control diabetic group (Fig. 5C).

### LTF treatment attenuated microvascular damage in the retina of diabetic rats

**Fig. 6:**
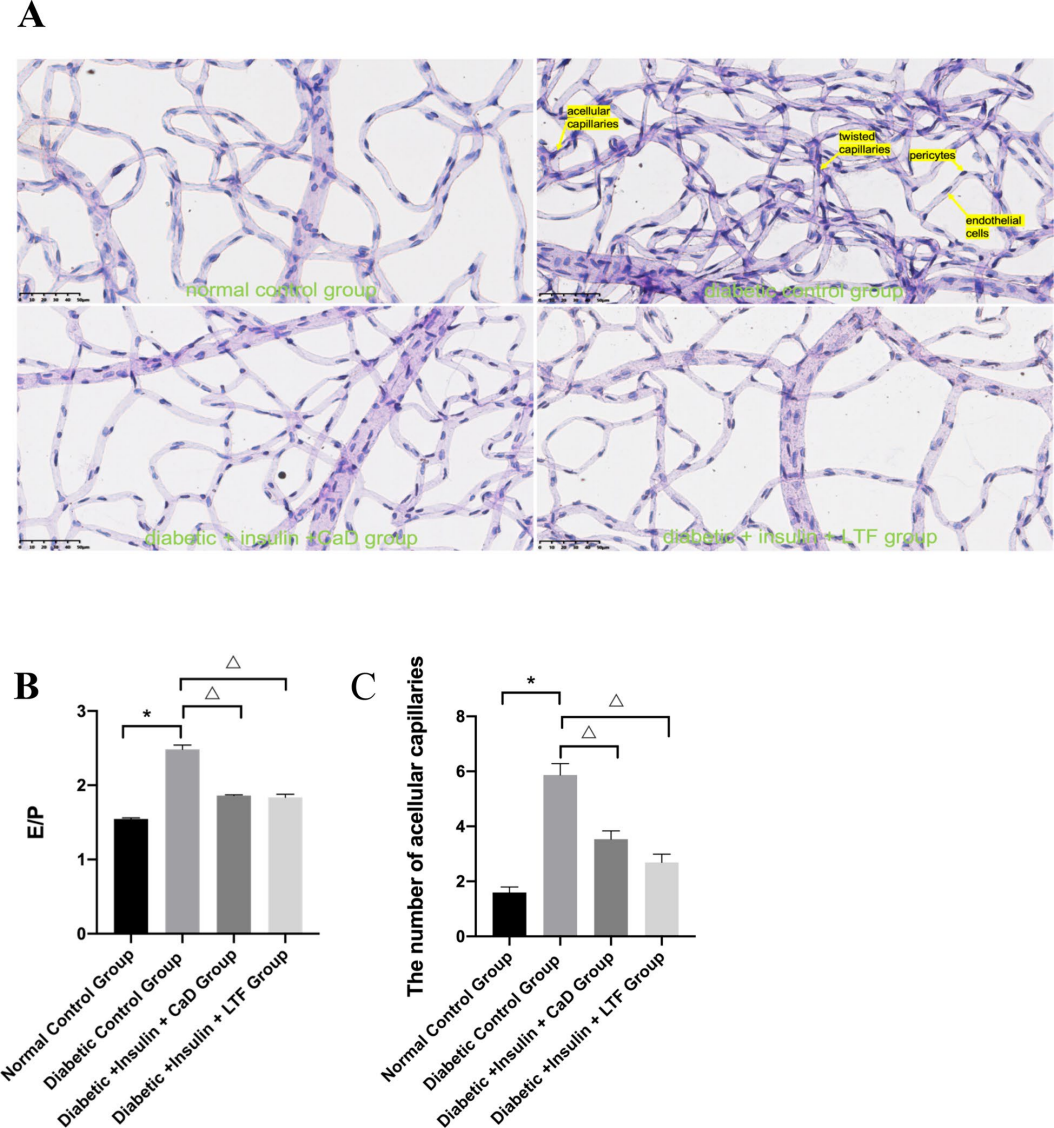
Effect of LTF and CaD on the microvascular damage in the retina. **A** PAS staining of retina (×400). **B** The ratio of endothelial cells and pericytes. **C** The number of acellular. Data are expressed as mean ± SD (n = 3) (*p < 0.05: vs. normal control group; ^*△*^*p* < 0.05: vs. diabetic control group)

Acellular capillaries, endothelial cells, and pericytes were studied using a retinal trypsin digestion assay. Diabetic control rats displayed twisted capillaries, more acellular capillaries, and a higher ratio of endothelial cells to pericytes (E/P) compared to those observed in normal rats. CaD as well as LTF treatments dramatically reduced the number of twisted capillaries, acellular capillaries, and the E/P ratio (Fig. 6).

### LTF treatment activated cell junction protein expression in the retina

**Fig. 7:**
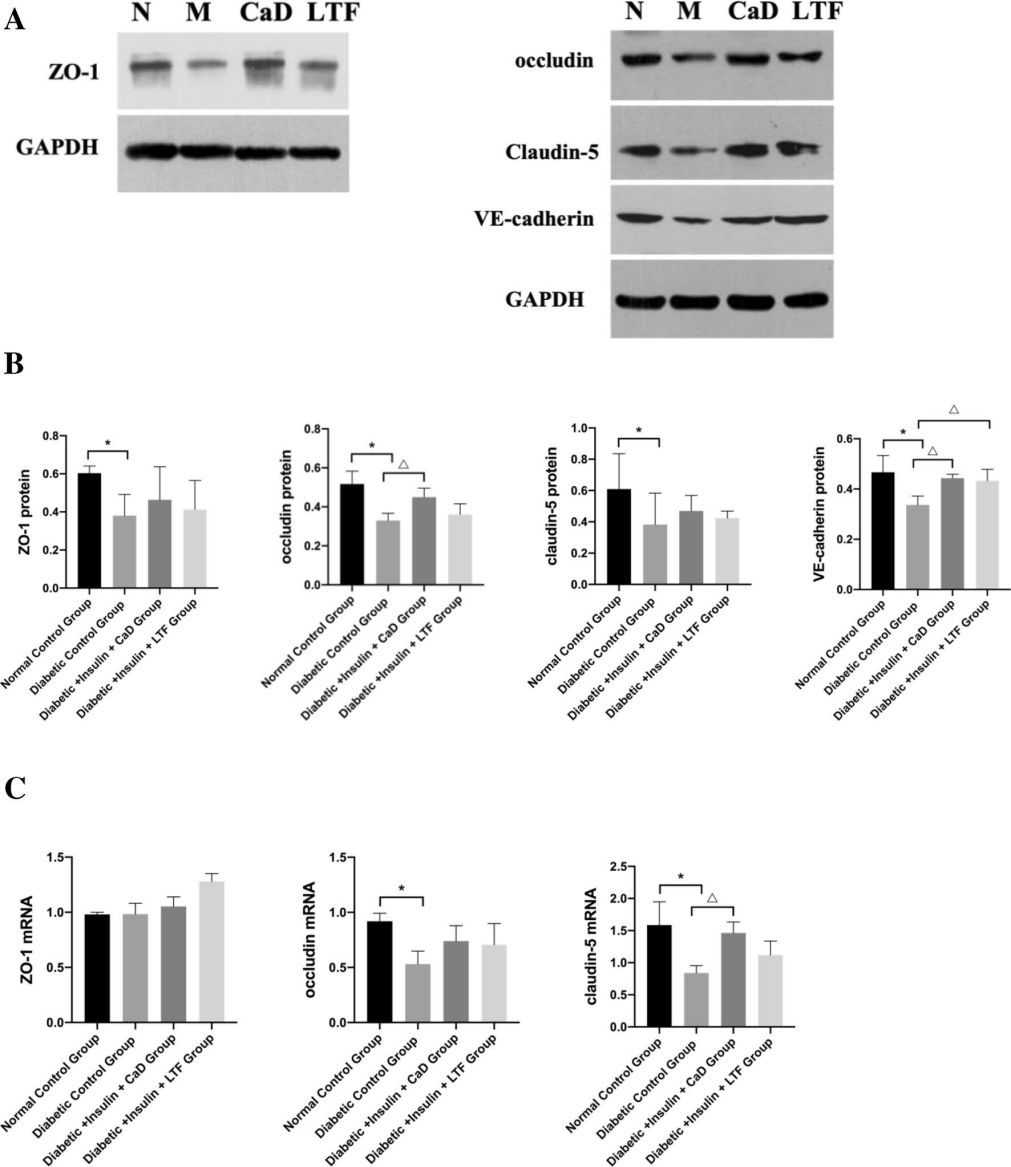
Effect of LTF and CaD on the cell junctions expression in the retina. **A**, **B** Western blot and quantitative measurement of retinal ZO-1, occludin, claudin-5, and VE-cadherin. **C** Real-time PCR analysis of ZO-1, occludin, and claudin-5. Data are expressed as mean ± SD (n = 3) (*p < 0.05: vs. normal control group; ^△^*p* < 0.05: vs. diabetic control group)

The protein levels of ZO-1, occludin, claudin-5, and VE-cadherin in the diabetic controls were significantly elevated. Compared with the diabetic control group, the CaD treatment notably increased protein levels of occludin and VE-cadherin, while there were no significant differences in ZO-1 and claudin-5 protein levels. The LTF treatment significantly increased VE-cadherin protein levels; however, there was no significant difference in ZO-1, occludin, and claudin-5 protein levels compared to those observed in diabetic control rats (Fig. 7A, B). Moreover, occludin and claudin-5 mRNA levels were significantly elevated in diabetic control animals, while CaD and LTF treatments increased the mRNA levels of ZO-1, occludin, and claudin-5 to varying degrees (Fig. 7C).

### LTF treatment altered gut microbiota in a rat model

**Fig. 8:**
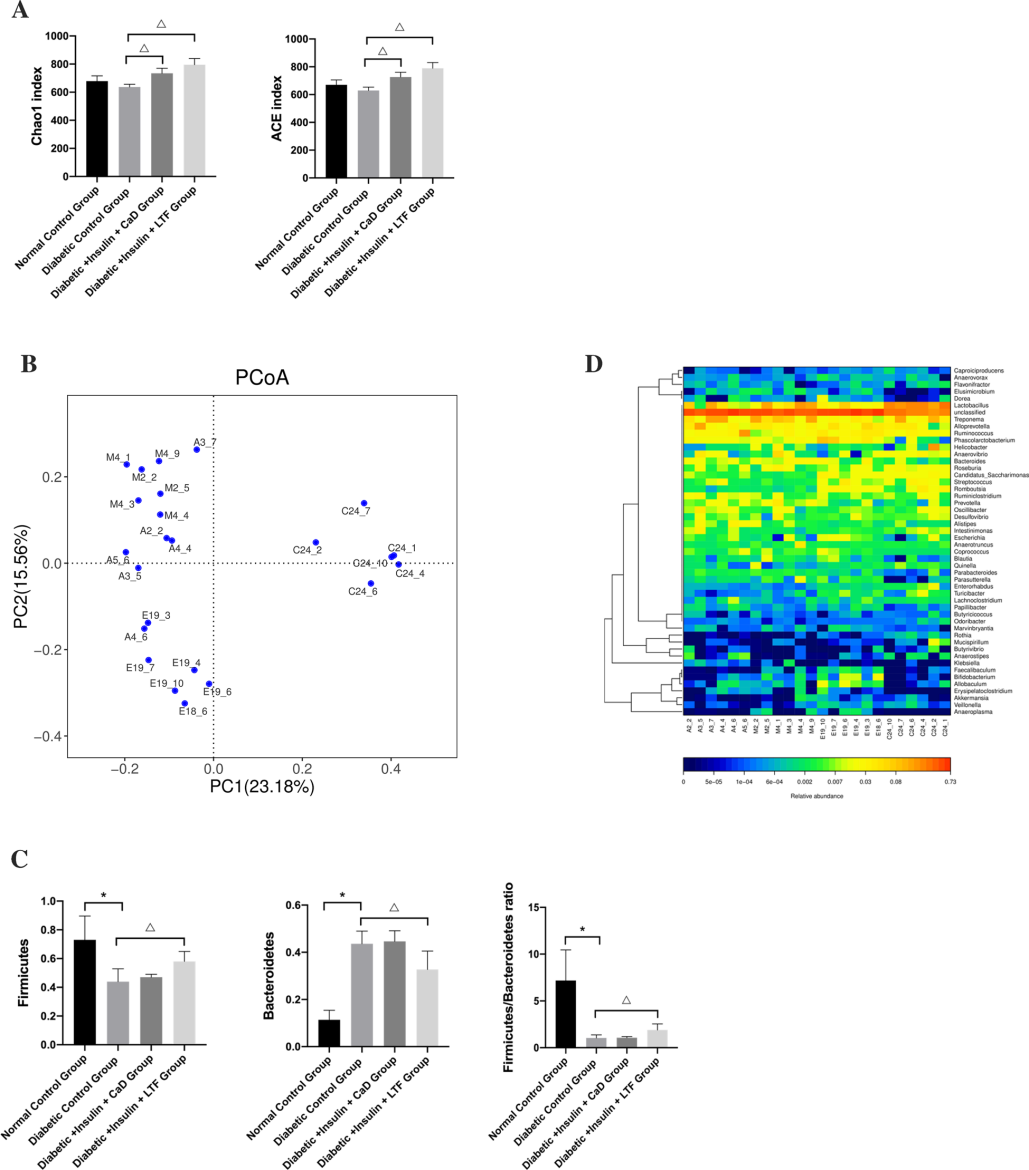
Effect of LTF and CaD on gut microbiota in a rat model. **A** The α diversity of the gut microbiota. **B** Bray–Curtis distance PCoA. **C** The levels of *Firmicutes* and *Bacteroidetes*, and the ratio between the two bacteria. **D** Heatmap represents the hierarchical clustering based on the microbial composition at the genus level. Data are expressed as mean ± SD (n = 6) (*p < 0.05: vs. normal control group; ^△^*p* < 0.05: vs. diabetic control group)

To analyze changes in the gut microbiota, we performed Illumina sequencing of V3 and V4 regions of the *16S rRNA* gene using stool samples obtained from different groups. A total of 830,120 quality sequencing reads were obtained, with an average of 34,588 sequencing reads per sample. In total, 874 OTUs (> 97% similarity) were identified. The α-diversity of the intestinal microbiota indicated that both CaD and LTF significantly increased ACE richness and Chao’s diversity index (*p* < 0.001) when compared with the intestinal microbiota of diabetic control animals (Fig. 8A). PCoA based on Bray–Curtis distance showed that the LTF treatment significantly changed the gut microbiota of diabetic rats, indicating that core microbiota had undergone significant LFT-induced changes (Fig. 8B). These results demonstrated that LTF restored the intestinal microbiota of diabetic rats to a level comparable with that of normal rats.

In addition, the differences in phylum and genus levels among different experimental groups were explored. At the phylum level, two major phyla were distinctly altered in diabetic rats, as shown by a decrease in *Firmicutes* and an increase in *Bacteroidetes*, compared to normal rats. Following 12 weeks of LTF treatment, a significant increase in *Firmicutes* levels, a decrease in *Bacteroidetes* levels, and an increase in the ratio of *Firmicutes* to *Bacteroidetes* was observed, changes that were not significantly different from those observed in the CaD group (Fig. 8C). At the genus level, a closer examination showed that the abundance of *Candidatus_Saccharimonas*, *Romboutsia*, *Enterorhabdus* in the LTF group was higher than that in the diabetic control, with *Romboutsia*, and *Enterorhabdus* significantly enriched. *Prevotella* in the LTF group was significantly lower than that in the diabetic control group, while the reduction of *Anaerotruncus* in LTF group was not significant. However, no significant changes were observed in the CaD group (Fig. 8D). These data indicated that the LTF treatment changed gut microbiota structure and altered the genera.

### LTF treatment attenuated systemic and local retinal inflammation

**Fig. 9:**
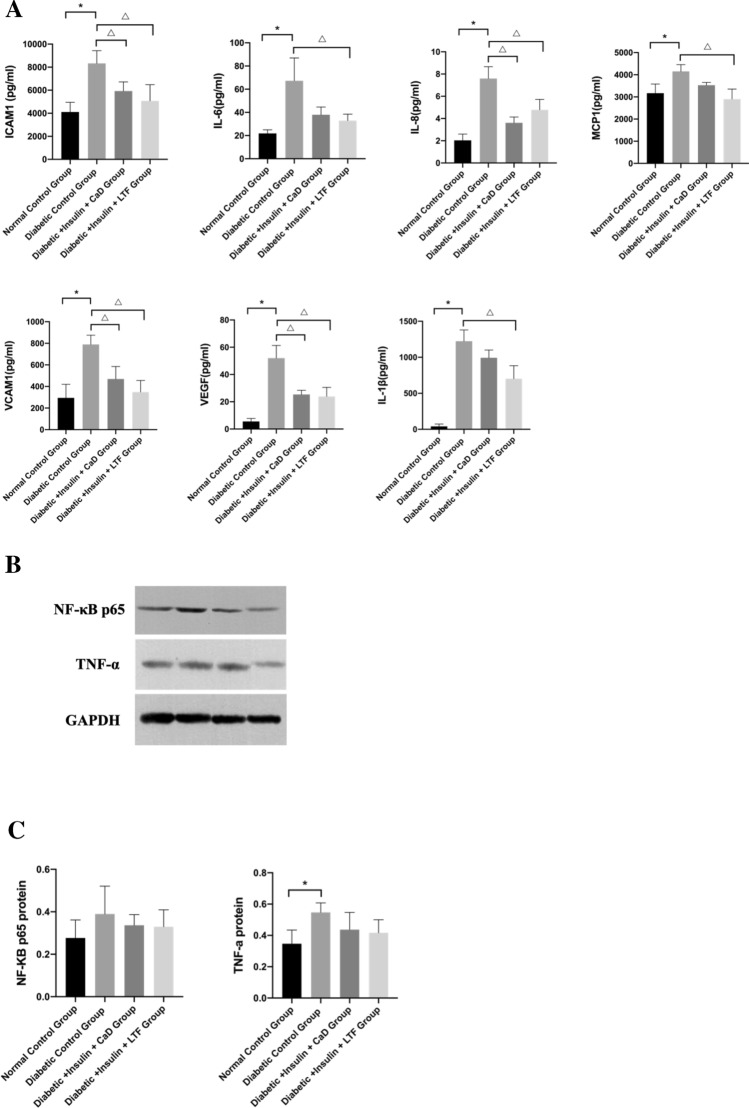
Effect of LTF and CaD on systemic and local retinal inflammation. **A** Elisa measurement of proinflammatory cytokines in serum, including ICAM-1, IL-6, IL-8, MCP-1, VCAM-1, VEGF, and IL-1β. Data are expressed as mean ± SD (n = 6). **B**, **C** Western blot and quantitative measurement of retinal NF-κB and TNF-α. Data are expressed as mean ± SD (n = 3) (*p < 0.05: vs. normal control group; ^△^*p* < 0.05: vs. diabetic control group)

To determine whether inflammation in the diabetic control group was improved, we measured the levels of several inflammatory factors in the serum and tissues of different groups. Serum levels of ICAM-1, IL-6, IL-8, MCP-1, VCAM-1, VEGF, and IL-1β were significantly increased in the diabetic control group compared with the normal control group. Our results indicated that four pro-inflammatory cytokines, ICAM-1, IL-8, VCAM-1, and VEGF in the sera obtained from the CaD group were significantly decreased compared to those measured in sera of diabetic control rats. The levels seven pro-inflammatory cytokines, ICAM-1, IL-6, IL-8, MCP-1, VCAM-1, VEGF, and IL-1β in the LTF group were significantly decreased compared to those measured in sera of diabetic control rats (Fig. 9A). We also discovered that the protein levels of NF-κB and TNF-α were elevated in diabetic control rats compared with those in normal control rats, with the significant difference in TNF-α. NF-κB and TNF-α were decreased in the retinas of the CaD and LTF treated animals; however, these differences were not statistically significant (Fig. 9B, C). Considered together, our results revealed that systemic and local retinal inflammation was alleviated via LTF treatment.

### Gut microbiota associated with inflammation improvement in DR

**Fig. 10:**
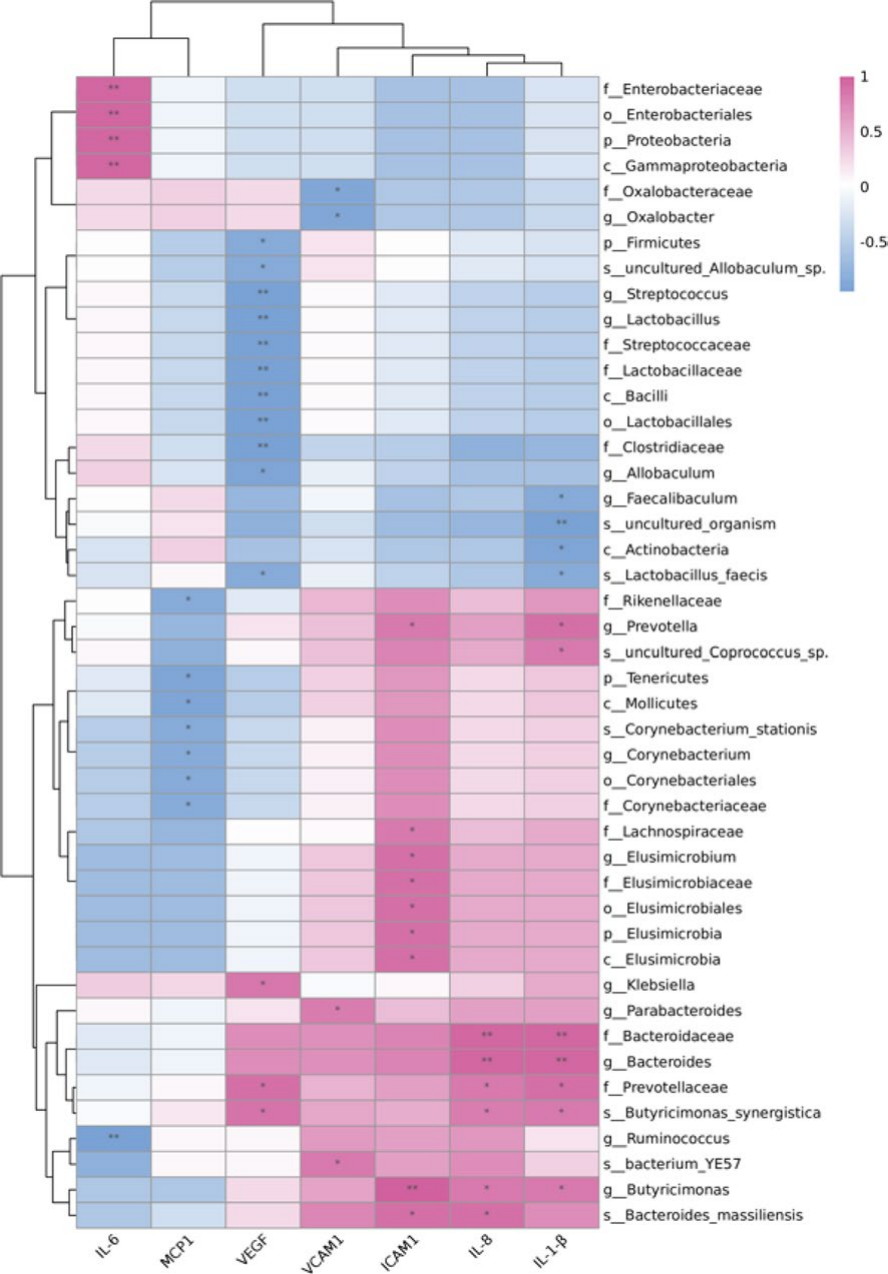
Association of the vital gut microbiota changed by LTF with improvements in proinflammatory cytokines. Blue represents negative correlation, and red represents positive correlation (*: 0.01 < *p* < 0.05; **: 0.001 < *p* < 0.01)

Further, we explored key gut microbiota that was correlated with the effect of LTF on inflammatory cytokines, including ICAM-1, IL-6, IL-8, MCP-1, VCAM-1, VEGF, and IL-1β. Of the gut microbiota in the diabetic control group that were altered following LTF treatment, *Enterobacteriales*, *Prevotellaceae*, *Enterobacteriaceae*, *Bacteroides*, and *Klebsiella* showed significant positive correlations with the inflammatory factors associated with DR. Our results revealed positive correlations between *Enterobacteriales* and IL-6, *Prevotellaceae* and IL-8, VEGF, as well as IL-1β, *Enterobacteriaceae* and IL-6, *Bacteroides* and IL-8 as well as IL-1β, and *Klebsiella* and VEGF. Alternatively, *Faecalibaculum* and *Corynebacterium* were negatively correlated with the inflammatory factors such as IL-1β and MCP-1, respectively. Considered together, LTF significantly enriched *Faecalibaculum* and *Corynebacterium* and decreased the abundance of *Enterobacteriales*, *Prevotellaceae*, *Enterobacteriaceae*, *Bacteroides*, and *Klebsiella*; thus indicating a significant correlation between gut microbiota and the mechanism through which LTF facilitated the alleviation of DR-induced inflammation (Fig. 10).

## Discussion

LTF, which consists of *Radix Rhei Et Rhizome*, *Persicae Semen*, *Panax notoginseng (Burk.) F. H. Chen Ex C. Chow*, and *Hirudo* has been used to treat diabetic secondary symptoms, such as DR, diabetic nephropathy, diabetic encephalopathy, and diabetic cardiomyopathy [16–19]. Previous studies from our lab have shown that LTF ameliorates DR via certain mechanisms, involving the miR-200b and the p38 MAPK/NF-κB pathway [13, 14]. Nevertheless, the impact of LTF on the intestinal microbiota of diabetic rats, as well as the impact of altered microbiota on the host remains unclear. Based on one of our previous studies, we hypothesized that LTF may modulate the gut microbiota of diabetic rats, which in turn might reduce inflammation. Our results indicated that LTF distinctly attenuated retinal damage, including retinal histology and BRB microvascular damage, while increasing tight junction protein expression and modulating gut microbiota, which in turn ameliorated systemic and local retinal inflammation.

Following 12 weeks of treatment with CaD or LTF, slightly decreased HbA1c and FBG levels were observed in diabetic rats, suggesting that both CaD and LTF prevented and delayed DR without apparently ameliorating hyperglycemia. This is consistent with our previous findings which indicated that herbs promoting blood circulation and preventing blood vessel blockage may delay the occurrence and development of microvascular complications in diabetes without apparently ameliorating hyperglycemia [20]. Xiaolin Tong, a member of the Chinese Academy of Sciences, stated that diabetes, a collateral blood glucose disease, can be defined as a collateral injury caused by hyperglycemia. Thus, curing diabetes-induced collateral injuries—including during its early stages—is vital. LTF is derived from the herb Di Dang Tang, which activates blood and resolves stasis, according to *Shang Han Lun*, a medical treatise compiled during the East Han Dynasty. Our previous studies have shown that herbs that promote blood circulation and remove obstruction in collateral injuries can alleviate fundus lesions and reduce microalbumin in the urine of diabetic patients. Additionally, these herbs reduced and delayed the occurrence and development of diabetic microangiopathy in diabetic rats, by repairing vascular damage and attenuating vascular leakage in the retina [13, 20, 21].

To confirm whether LTF has an effect on the retinal histology of diabetic rats, we examined related indicators such as fundus oculi, retinal thickness, as well as microvascular and BRB permeability. Our FFA examination revealed yellowish-white spots, namely hyperfluorescence, contiguous patches, retinal veins with widened diameters, and diabetic ocular fundi exhibiting smaller arteriovenous ratios. Fundus oculi damage was effectively attenuated by CaD and LTF treatment. BRB permeability increased with high glucose levels, and although LTF significantly decreased BRB permeability compared to that observed in diabetic control rats, no significant decrease was observed in the CaD group. We also observed that retinal thickness was reduced in diabetic rats, and that both CaD and LTF significantly increased retinal thickness. Furthermore, acellular capillaries and the E/P ratio were increased in diabetic rats, whereas these two indicators were attenuated following CaD and LTF treatment. These results indicate that, although both CaD and LTF ameliorated retinal pathological damage, the effect exerted by LTF was superior to that exerted by CaD. This is in line with the results of our previous studies showing that LTF inhibits pathological changes in diabetic rats. Collectively, these findings clearly show that LTF plays an important role in protecting BRB and reducing pathological damage [13].

Cell junctions, including tight junction proteins (e.g., occludin and claudins) and adherens junctions (e.g., VE-cadherin) strictly regulate the fluids, solutes, and cells that cross the BRB. A characteristic sealant ring, consisting of tight junction proteins and adherens junctions along the apical or basal perimeter of cells, restricts the access via paracellular routes. Peripheral proteins (e.g., ZO-1) may modulate the initial formation of tight junction proteins, while cell junctions and peripheral proteins regulate BRB and retinal vascular permeability [22]. Protein levels, including ZO-1, occludin, claudin-5, and VE-cadherin, as well as occludin and claudin-5 mRNA levels were significantly elevated in diabetic controls. CaD and LTF increased these indicators to varying degrees. These results indicated that LTF prevents BRB via a mechanism which enhances the expression of cell junctions and peripheral proteins.

Recent studies have shown that the gut microbiome is involved in the development as well as treatment of DR [5, 23–26]. However, only a few TCM studies have described the potential benefits of gut microbiota alteration as a therapeutic strategy against DR. Here, we observed obvious gut microbiota alterations in diabetic rats compared with normal rats. In diabetic rats, the α-diversity of the gut microbiota, including ACE richness and Chao’s diversity index, were significantly decreased. Meanwhile, PCoA based on Bray–Curtis distances indicated that vital microbiota at the phylum and genus levels, such as *Firmicutes* and *Bacteroidetes*, were distinctly altered in diabetic rats compared to those observed in normal rats. Murri et al. [27] showed that children with T1DM displayed increased *Bacteroidetes* as well as decreased *Firmicutes* and *Prevotell*a levels. Gao et al. [28] and Yanni et al. [29] discovered that *Romboutsia* was decreased in T1DM rats. These changes in the microbiota were consistent with our results. After 12 weeks of treatment, both CaD and LTF distinctly increased ACE richness and Chao’s diversity indices. Interestingly, LTF significantly changed the PCoA based on Bray–Curtis distance of the microbiota at the phylum level, increasing *Firmicutes* and decreasing *Bacteroidetes*. Furthermore, the LTF treatment also affected vital microbiota at the genus level such as *Romboutsia*, *Enterorhabdus*, and *Prevotell*a, which were not significantly altered in the CaD group. Wei et al. [30] suggested that sennoside A, the main active ingredient of rhubarb, elevated the levels of *Firmicutes* and reduced the levels of *Bacteroidetes*, which are associated with intestinal permeability and inflammation, in diabetic rats. These data suggest that LTF may ameliorate DR mainly via the modulation of gut microbiota.

To further investigate whether LTF treatment alleviates inflammation in diabetic rats, several inflammatory markers from serum and tissues were measured. We also analyzed the correlation between inflammation and the microbiome. We discovered that pro-inflammatory cytokines including ICAM-1, IL-6, IL-8, MCP-1, VCAM-1, VEGF, and IL-1β from the serum of diabetic rats, as well as NF-κB and TNF-α in the retina, were all increased. Four cytokines were significantly decreased following the CaD treatment, and seven were decreased following the LTF treatment; thus, suggesting that LTF effectively alleviated systemic inflammation. There was a positive correlation between IL-6, IL-8, IL-1β, and VEGF levels, which were significantly decreased by the LTF treatment, and several microorganisms, including *Enterobacteriales*, *Enterobacteriaceae*, *Prevotellaceae*, *Bacteroides*, and *Klebsiella*, which were altered by the LTF treatment. Yan et al. [31] reported that *Lactobacillus acidophilus* KLDS1.0901 supplementation decreased the abundance of *Bacteroides* and lowered pro-inflammatory cytokines such as IL-8, IL-1β, and TNF-α, in diabetic rats. Furthermore, *Prevotella* has been shown to be positively correlated with IL-6, IL-8, and TNF-α in diabetic rats [32]. Moreover, a previous study showed that *Bacteroides vulgatus, Bacteroides rodentium*, and *Bacteroides xylanisolvens* abundances as well as IL-6 levels were significantly increased in T1DM patients [33]. Overall, these data indicate that LTF alleviated inflammation in diabetic rats by modulating gut microbiota.

Our study was affected by several limitations. First, only the overall therapeutic effect of LTF against DR, rather than that of its individual components, was evaluated. The main chemical components of LTF that contribute to the prevention and cure of DR were not explored in this study. The main compounds in LTF, such as Notoginsenoside R1 derived from *Notoginseng radix et rhizoma*, have been shown to play an important role in ameliorating diabetic retinopathy [34]. Several studies have shown that amygdalin, aloe-emodin, oleanolic acid, and rhein identified in LTF exerted an anti-inflammatory role in many diseases [35–40]. Thus, further studies aimed at clarifying the exact roles of the main LTF components are necessary. Lastly, the SCFA levels in the feces from animals of different groups were not measured. The study focused on the alteration in diversity and structure of gut microbiota, whereas the interactions between microbiota (such as antagonism) have not been explored in depth.

In conclusion, we revealed the mechanisms underlying the therapeutic effects of LTF against DR development and progression. Our findings indicate that the therapeutic formula of LTF, which is intended for promoting blood circulation and removing obstructions in collateral injuries, distinctly attenuates retinal damage during the early stages of diabetes. Our results also suggest that LTF potentially attenuates systemic and local retinal inflammation by regulating gut microbiota. Finally, our study provides reliable evidence that LTF may ameliorate DR during the early stage of DM via the “microbiota–inflammation–retina” axis.

## Data Availability

Please contact the author for data requests.

## Abbreviations

DR: Diabetic retinopathy
LTF: Luo Tong formula
GC–MS: Gas chromatography–mass spectrometry
LC–MS: Liquid chromatography–mass spectrometry
FBG: Fasting blood glucose
IDF: International Diabetes Federation
DM: Diabetes mellitus
BRB: Blood-retinal barrier
TNF-α: Tumor necrosis factor-alpha
IL-1β: Interleukin-1β
IL-6: Interleukin-6
IL-8: Interleukin-8
NF-κB: Nuclear factor-κB
ICAM-1: Intercellular adhesion molecule-1
VCAM-1: Vascular cell adhesion molecule-1
MCP-1: Monocyte Chemoattractant Protein-1
VEGF: Vascular endothelium growth factors
CaD: Calcium dobesilate
ESI: Electrospray ionization
FP: Fundus photography
FFA: Fundus fluorescein angiography
EB: Evans blue
OS: Outer segment
ONL: Outer nuclear layer
OPL: Outer plexiform layer
INL: Inner nuclear layer
IPL: Inner plexiform layer
GGL: Ganglion cell layer
E/P: Endothelial cells to pericytes
ANOSIM: Analysis of Similarity
PCoA: Principal Coordinate Analysis
TCM: Traditional Chinese medicine
